# SNOMED CT standard ontology based on the ontology for general medical science

**DOI:** 10.1186/s12911-018-0651-5

**Published:** 2018-08-31

**Authors:** Shaker El-Sappagh, Francesco Franda, Farman Ali, Kyung-Sup Kwak

**Affiliations:** 1 Information Systems Department, Faculty of Computers and Informatics, Benha University, Banha, Egypt; 2 Department of Philosophy, University at Buffalo, Buffalo, NY USA; 3 Department of Information and Communication Engineering, Inha University, Incheon, South Korea

**Keywords:** SNOMED CT, Ontology, Clinical terminology, Electronic health records, Description logic

## Abstract

**Background:**

Systematized Nomenclature of Medicine—Clinical Terms (SNOMED CT, hereafter abbreviated SCT) is a comprehensive medical terminology used for standardizing the storage, retrieval, and exchange of electronic health data. Some efforts have been made to capture the contents of SCT as Web Ontology Language (OWL), but these efforts have been hampered by the size and complexity of SCT.

**Method:**

Our proposal here is to develop an upper-level ontology and to use it as the basis for defining the terms in SCT in a way that will support quality assurance of SCT, for example, by allowing consistency checks of definitions and the identification and elimination of redundancies in the SCT vocabulary. Our proposed upper-level SCT ontology (SCTO) is based on the Ontology for General Medical Science (OGMS).

**Results:**

The SCTO is implemented in OWL 2, to support automatic inference and consistency checking. The approach will allow integration of SCT data with data annotated using Open Biomedical Ontologies (OBO) Foundry ontologies, since the use of OGMS will ensure consistency with the Basic Formal Ontology, which is the top-level ontology of the OBO Foundry. Currently, the SCTO contains 304 classes, 28 properties, 2400 axioms, and 1555 annotations. It is publicly available through the bioportal at *http://bioportal.bioontology.org/ontologies/SCTO/*.

**Conclusion:**

The resulting ontology can enhance the semantics of clinical decision support systems and semantic interoperability among distributed electronic health records. In addition, the populated ontology can be used for the automation of mobile health applications.

## Background

Clinical terminology is “a representational artifact containing a list of lexical entities, complete with definitions, used in some domain and formulated in a natural language” [1]. The goal of standardized clinical terminology such as Systematized Nomenclature of Medicine—Clinical Terms (SNOMED CT, hereafter abbreviated SCT) is to create a taxonomy of terms referring to entities in a given medical environment [2–8] and a framework of rules guaranteeing that each term is used with exactly one meaning; each meaning salient in the environment is expressed using exactly one term [9]. Each term in this taxonomy is in one or more parent–child relationships to some other terms in the taxonomy. For a full definition and examples of clinical terminologies, readers are guided to Ivanovic and Budimac [10]. SCT is not compliant with any formal upper-level ontology, and it allows for multiple inheritances, which causes a messy situation in the classification of entities [11]. SCT has been implemented in a variety of operational systems, including electronic health record (EHR) semantic queries, cross mapping, and clinical decision support systems (CDSSs) [2]. However, SCT is still normally distributed as pipe (“|”)-separated text files [3]. These files are used to encode and retrieve medical data using text-based matching. The structure and the expressiveness of the SCT underlying formalism has not changed significantly since the mid-1990s. On the other hand, there have been significant developments in both logic-based formalisms and ontology design since then.

### SNOMED CT and the ontology

An ontology can solve many challenges in the SCT structure and semantics [12]. It is a formal and explicit representation of a shared conceptualization [13]. More formally, an ontology (O) is defined as: *O* = *TBOX* + *ABOX*. In this expression, *TBOX*=(*C*, ≤_*C*_, *R*, *σ*_*R*_, ≤_*R*_, *A*, *σ*_*A*_, *T*) is the ontology terminology, where *C*, *R*, *A*, and *T* represent disjoint sets of concepts, relations, attributes, and data types; ≤_*C*_ is the taxonomy or concept hierarchy; ≤_*R*_ is the relations’ hierarchy; *σ*_*R*_ : *R* ⟶ *C*^+^ represents the relations’ signatures, which define what concepts are involved in one specific relation of the set *R*; and *σ*_*A*_ : *A* ⟶ *C* × *T* is the signature of an attribute of a certain concept, *C*, which takes values of a certain data type T. And *ABOX* is the ontology instantiation in the form *C*(*I*_1_), with *R*(*I*_1_, *I*_2_) for *I*_*j*_ as the ontology instances. From this definition, the terminology can be considered a lightweight ontology with low semantics. Ivanovic and Budimac [10] defined medical terminologies and vocabularies, and compared them with ontology semantics.

he large size of SCT makes defining new terms and maintaining the existing collection a challenging task [14]. According to Zhang et al. [15], SCT data released to third parties do not have integrity constraints between relationships in the release data, in the database sense of the term. In other words, there are no optionality/mandatory constraints, and cardinality is always many-to-many, creating a completely unconstrained model; any system implementation will have to create its own integrity maintenance.

Bodenreider et al. [16] evaluated the consistency of SCT with seven ontology principles, such as subsumption and a hierarchical structure according to a description logic (DL) perspective, and their study shows that the current form of SCT has many limitations. For example, many classes have only a single child, such as {*Multiple polyps*, child: *Multiple adenomatous polyps*} (*morphologic abnormality*), whereas some other classes have an unusually large number of children, such as *Oxidoreductase (substance) (571 children*). Children must be different from their parents. It is common to find properties or relationships specific to the parent class not being inherited by the children, as for example in the case of the parent class *Subjective visual disturbance*, which is described as having possible clinical courses of *sudden onset* or *gradual onset*; but the child *sudden visual loss* has *sudden* as its only valid *onset*. Given that each term in this taxonomy is in one or more parent–child relationships to some other terms in the taxonomy, another problem related to the hierarchical structure of SCT is multiple inheritances, which causes confusion in classification of the entities [11].

In addition, SCT exploits its taxonomy structure for classifying concepts, but it has an uncontrolled use of IS_A to signify a variety of different types of relations (such as PART_OF, IS_A_INSTANCE_OF, and so on), which results in IS_A overload and incorrect subsumption. Dentler and Cornet [17] discovered that 35,010 concepts (12%) contained redundant elements in their definitions in the July 2012 version of SCT. Other issues detected with SCT are false synonymies, failure in the use-mention distinction, and “incoherent ontological commitment” [18]. According to Bodenreider et al. [16], compliance with sound ontological principles would guarantee the accuracy of reasoning based on SCT.

### SNOMED CT and the database

Many efforts have been made to enhance the SCT structure and semantics by using a database technology [15, 19]. But Campbell et al. [20] asserted that SCT databases have limitations, including reduction of data richness, limitations on the query capability, and increased systems overhead. A database converts the SCT main text-based data files (i.e. concepts, descriptions, and relationships) into three interrelated database tables based on CONCEPT_IDs [19]. As a result, the SCT knowledge base is ∑ = (T, A), where T is the schema, and A represents the instances. Structured Query Language (SQL) queries on ∑ can be used to fetch specific concepts, descriptions, or relationships that are used for medical data encoding, natural language processing, and building a user interface in EHR ecosystems [21]. Regarding intelligence and inference capabilities, databases are weak in making inferences, because databases support queries only based on explicitly stated data instances, and they are based on a closed-world assumption (CWA) [22, 23]. CWA assumes that ∑ contains complete data. Based on ∑, if a SQL query asking for fact C did not find it explicitly stated, then a clear result will be returned (0 or NULL). This concept is known as negation as failure (NaF) or (NOT TRUE = FALSE), or ∑⊭C. The resulting database models for SCT cannot be utilized to answer semantic queries, and no reasoner has been used to make inferences from databases. Some studies, such as the one by Schadow et al. [24], implemented the logical model of SCT in relational database format by transitive closure Table (TC), which represents only subsumptive relationships in the form < Ancestor, Descendant>. However, this limited table requires extensive recursive calculations. For example, the TC table for the 2014 SCT exceeds five million rows [20]. In its application programming interface (API) called Snofyre [25], National Health Services proposed an SCT object model where SCT contents are implemented as Java classes, but this object model still lacks inference capabilities.

An ontology has more advantages than a database, because it is based on a formal description logic and open-world assumption (OWA) [22, 23]. The ontology knowledge base is ∑ = (T, A), where T = TBOX, and A = ABOX. Based on the formal DL, reasoners infer hidden knowledge and add it to ∑. OWA assumes that the ∑ contains incomplete knowledge, so no existing knowledge means “NOT KNOWN.” For example, if ∑ does not include knowledge about a patient’s allergy, it is not correct to say that the patient does not suffer from an allergy (i.e. NOT TRUE = UNKNOWN). Additional information is required to confirm or refute this hypothesis. A negative answer (i.e. NaF) is returned if the query contradicts other axioms in ∑. There are many reasoners and tools in the literature to support ontology engineering and semantic reasoning [26]. As a result, thanks to formal ontology principles, converting this terminology into an ontology enhances the formal logic–based inference (e.g. subsumption and equivalence) capabilities, redundancy checking, semantic consistency checking, and the definition of formal logic–based semantics for defining concepts. Campbell et al. [20] asserted that access to the full logical model of SCT is necessary. Souvignet et al. [27] and Schulz et al. [28] asserted that although SCT was not developed using OWL DL standard language, its general structure and formalism could be converted into an ontological representation by using EL++ DL^1^^,^^2^ [29].

### SNOMED CT and top-level ontologies

The ontological foundations of the large clinical terminology in SCT have substantially evolved; the International Health Terminology Standards Development Organization (IHTSDO) [3] and Gao and Khazai [30] showed how SCT conforms to the OWL standards. However, there are no upper-level ontologies to describe the SCT concept model (SCM) [31], i.e. formal or semiformal systems of categories, relationships, and axioms. Héja et al. [32] and Lopez-Garcia and Schulz [33] asserted that the SCT ontology (SCTO) is error prone without alignment with highly constrained and formal upper-level ontologies, such as BFO, Descriptive Ontology for Linguistic and Cognitive Engineering (DOLCE), or BioTopLite [1, 31, 34]. Rodrigues et al. [35] asserted that to build a common ontology, you have to agree on a common model of meaning. Although SCT is published twice a year (January and July) in Release Format 2 (RF2), the 19 top-level concepts are not changed between these releases; new releases mainly try to fix redundancies, inconsistencies, errors, and shortages [6, 36]. For each new release, IHTSDO provides textual release notes and delta files to determine the atomic additions and removals of concepts and relationships [7]. However, SCT top-level concepts or categories, which are formally modeled by the SCM, are stable. The Open Biomedical Ontologies (OBO) Foundry advocates the use of an upper-level ontology [37]. Using a common upper-level ontology for building the SCTO has several advantages, such as

1. forced categorization of domain entities into well-defined upper-level categories;
2. standardized ways in which entities are related to each other through a well-defined canon of relations;
3. better interoperability with other heterogeneous semantic resources and biomedical ontologies in the future; and
4. support for coordination between the structure of the terminology and medical records, which is one of the requirements that Cimino [38] and Rector [39] mentioned.

There are many existing biomedical top-level ontologies, such as the Ontology for General Medical Science (OGMS), BioTop, BioTopLite, the Basic Formal Ontology (BFO), etc. [34, 40, 41]. OGMS extends BFO [42]. Most of the OBO Foundry ontologies are aligned with BFO, which makes BFO the best choice for building the SCT upper-level ontology [31, 41]. One of the main advantages of BFO is its *realism-based* approach, namely, the view that our thoughts, representations, beliefs, and knowledge are about reality [1]. Thus, an ontology is a *representational artifact* about the world itself and not concepts [43]. The problem arising from concept-based ontologies is that the term *concept* is often ambiguous [44]. Taking, for example, SCT itself, we know that *concept* can refer to 1) the clinical idea (for instance, the concept of kidney disease), 2) the ConceptId (a string, for example, “90,708,001”), and 3) the entity itself in the real world (the instances of kidney disease we actually find in patients’ bodies) [42]. Thus, the ambiguity often neglects the use-mention distinction. Resorting to the ontologically realistic perspective of BFO, the ambiguity is resolved: the terms in ontologies refer to universals, which are not concepts or strings, but mind-independent and repeatable features of reality where existence depends on the particulars (the concrete entities) by which they are instantiated [1, 16]. So, when we talk about *kidney disease*, we are sure we are talking about something in reality (namely, all the instances in the world of the kinds of kidney disease). Thus, we avoid use-mention mistakes, and we avoid incorrect conclusions (for example, that kidney diseases are concepts instead of real things in people’s bodies).

In order to build the SCT ontology, the SCT international release comes with a Perl transform script that converts the RF2 files into OWL format. This converter translates the concept hierarchy into an OWL taxonomy hierarchy by using the lightweight OWL EL description logic based on the *owl:subClassOf* property [3, 31]. To achieve balance between expressiveness of the used DL and its computation time, EL++ DL is commonly used in medical informatics [27–29] because it offers sufficient expressiveness and is computable within a reasonable time. According to the World Wide Web Consortium (W3C) guide for OWL 2, EL formalism “*is particularly suitable for applications employing ontologies that define very large numbers of classes and/or properties, captures the expressive power used by many such ontologies, and for which ontology consistency, class expression subsumption, and instance checking can be decided in polynomial time*.” EL++ DL will be discussed in Section “Discussion”; for more knowledge about it, see Dentler et al. [45] and Penaloza and Sertkaya [29].

Using EL++ DL supports its reasoners (e.g. CEL^3^) to compute the subclass hierarchy of a given ontology in polynomial time; however, the resulting OWL ontology is of such a big size (> 140 MB) that it runs into memory problems when loaded into most of the popular ontology tools [46]. Many projects on BioPortal are based on SCT, such as nursing, etc. [40]. We studied all of these ontologies and discovered that none of them have object and data properties, or axioms for post-coordination expressions. They are all lightweight taxonomies based on the *116,680,003|is a|* relationship. Recently, Schulz and Martínez-Costa [31] proposed a method for harmonizing SCT with BioTopLite [34]. However, this study has a critical limitation: it implemented the SCT relationships as object properties without preserving the hierarchical relationships between them. The semantics of the resulting ontology is not suitable for creating SCT post-coordinated expressions, because OWL does not support the creation of properties for object properties. Moreover, they used different terminologies to map SCT relationships to BioTopLite object properties, such as “has condition” to model a relationship between *clinical finding* and *procedure*.

The ultimate goal of this paper is to develop the SCTO upper level to manage and enforce the logical consistency of SCT concepts, descriptions, and relationships. This ontology is not populated with the SCT concepts, but that process is straightforward and will be handled in future research. The ontology designed here allows researchers to logically check the consistency and content coverage of SCT. Moreover, the proposed ontology can be used in EHR environments to provide data entry, information retrieval, and decision support capabilities in a more intelligent way based on the logical semantics of ontologies. The proposed ontology is built based on BFO as its top-level ontology [42, 44]. The SCTO is general enough so that it can be used as an overarching ontology for other domain-specific ontologies derived from SCT. OGMS is, in turn, based on BFO 2^4^ as the overarching top-level ontology [41, 42]. This binding process is critical in order to accomplish the following.

1. Identify a standard understanding of the SCT concepts’ semantic meanings. It forces the categorization of domain entities into well-defined upper-level categories connected with canonical relations. For instance, *allergy* can be a disposition or a process; *fracture* can be a damaged anatomical entity (the fractured bone) or a fracturing event. Ontology reasoners can detect inconsistencies and redundancies in the resulting ontology. This is helpful for SCT versioning, EHR coding, and terminology mapping [5, 11].
2. Build up logically coherent hierarchies. Using unified semantics for top-level classes prevents ambiguities. In addition, BFO and OGMS support only single inheritance, which can solve many inconsistencies that currently exist in SCT [1].
3. Solve the problem of the dynamic nature of post-coordinated concept definitions. The same complex concept can be represented with different post-coordinated concepts. Using unified OWL 2 axioms can define complex post-coordinated expressions in a unified and accurate way [3].
4. Facilitate the creation of CDSS systems using rule formats, such as Semantic Web Rule Language (SWRL), and rule engines such as Pellet, the Java Expert System Shell (JESS), or fuzzy JESS. SCTO’s TBOX and ABOX form a knowledge base. In addition, OWL 2 format supports the addition of IF-THEN rule axioms using SWRL. These rules can be utilized to build CDSSs for specific purposes based on all SCT semantics [47, 48]. In addition, semantic interoperability between distributed CDSSs and EHR systems is achieved.
5. Facilitate integration, harmonization, interoperability, data exchange, and mapping with other ontologies, like Gene Ontology (GO), International Classification of Disease, etc., because mapping is based on intelligent semantic similarity between classes, and not just lexical matching between the terms’ text [2, 5, 11, 19, 31, 49]. For a full list of supported mappings between SCT and other terminologies, readers can refer to Cardillo [50].

It is our hope that this study will provide a way for other researchers to use SCT for significant knowledge-engineering tasks. Our work is based on the 31/07/2015 release of SCT [3, 4, 30, 51].

## Methods

### SCT to OGMS mapping process

The mapping process is manually done for the 19 SCT top-level concepts according to the SCM [3]. First, we map each of the SCT top-level concepts to a specific class in OGMS [42]. Secondly, we use subClassOf and equivalentTo properties to build axioms to restrict the semantic meaning of each of these top-level universals. The mapping decision is made by analyzing the meaning of the candidate classes and relations, considering formal axioms as well as text definitions and hierarchical contexts of both OGMS and SCT classes. The mapping is iteratively checked by using some DL reasoners in the Protégé environment, such as Pellet. For identified inconsistent classes or axioms, we found a solution. In order to implement the SCTO, we used the Protégé 5 knowledge engineering tool,^5^ together with HermiT, Pellet, and FaCT++ ontology reasoners.

### Modeling pre-coordinated expressions

SCT expressions are defined as a structured combination of one or more concept identifiers used to express an instance of a clinical idea [3]. The expression consists of one or more focus concepts and optional refinements. To improve readability, SCT terms will be printed in italics rather than placed in single quotes. Clinical expressions in SCT concepts can be of two types: pre-coordinated expressions, which use a single SCT concept identifier, and post-coordinated expressions, which contain more than one SCT identifier. In pre-coordinated expressions, the clinical meaning of the expression matches the meaning of the unique listed concept, e.g. *73,211,009 |diabetes mellitus|*. Pre-coordinated concepts are identified by their defining relationships under the concept *246,061,005|attribute|*. Expressions will be represented using compositional grammar [9]. For example, diabetes mellitus is defined in SCT as follows.

Example 1

*73211009* |*Diabetes mellitus*| *<<< 126877002*|*disorder of glucose metabolism*|*, 362969004*|*disorder of endocrine system*|*, 363698007*|*finding site*| *113331007*|*structure of endocrine system*|*.*

where *<<<* is ⊑ and the comma represents *and*. Pre-coordinated concepts are determined in SCT concept files as either *primitive* (definitionStatusId = 900,000,000,000,074,008) concepts defined by subsumption operator⊑, or *fully defined* (definitionStatusId = 900,000,000,000,073,002) concepts defined by equivalence operator≡. Primitive concepts do not have unique relationships sufficient to distinguish them from their parents and sibling concepts. They only contain necessary relationships. In contrast, a fully defined concept has both necessary and sufficient relationships. Primitive concepts can only be used in post-coordinated expressions, whereas fully defined concepts can be used in pre-coordinated expressions. Users have to use a search engine, browser, or natural language processing to find the most suitable concept according to the concepts’ fully specified names (FSNs), synonyms, and contexts.

### Modeling of post-coordinated expressions

When the clinical idea is not stated on an “as is” basis in SCT, the user is able to compose already stated pre-coordinated concepts to form post-coordinated expressions [3]. SCM provides the rules that govern this process, and compositional grammar specifies the ways of this process [4, 9]. There are some main forms of post-coordination.

(1) The simplest form of a post-coordinated expression is the *combination* of multiple focus concepts. For example, *Needle biopsy of kidney* can be represented by an expression in compositional grammar.

Example 2

*Needle biopsy of kidney === 7246002*|*Kidney biopsy*|*, 129249002* |*Needle biopsy*|*.*

where the comma represents the conjunction of these two concepts, and the new concept *Needle biopsy of kidney* is equivalent (*===*) to this conjunction; but the two concepts on the right side must come from only one top-level hierarchy, which can be inferred or managed through OWL axioms. Expressions can be written without terms [4, 9] to make normal forms that are used to measure expression equivalence and subsumption. The previous expression can be written in the following normal form (the *equivalent to* sign [===] is the default, so it can be removed):

*129249002, 7246002*

(2) The most common form of post-coordination is the *refinement*, which is characterized by refining the value of one or more of the defining attributes of the concept through the use of the form *<attribute name* = *attribute value>*, as follows [3].

- The *attribute name* is a concept that is a subtype of *246,061,005*|*attribute*|.
- The refinement *attribute value* is a concept or expression that is appropriate to the attribute name as specified by the SCM. In most cases, any subtype child or descendant of a concept that is permitted as an attribute value of an attribute is also permitted as an attribute value.
- Refinements may be grouped to represent interdependencies between them in the same way as super-type relationship groups.

For example, *radius fracture* can be represented as:

===*125605004*|*Fracture of bone*|*: 363698007*|*Finding site*| *= 181940002*|*Radius*|

**Fig. 1:**
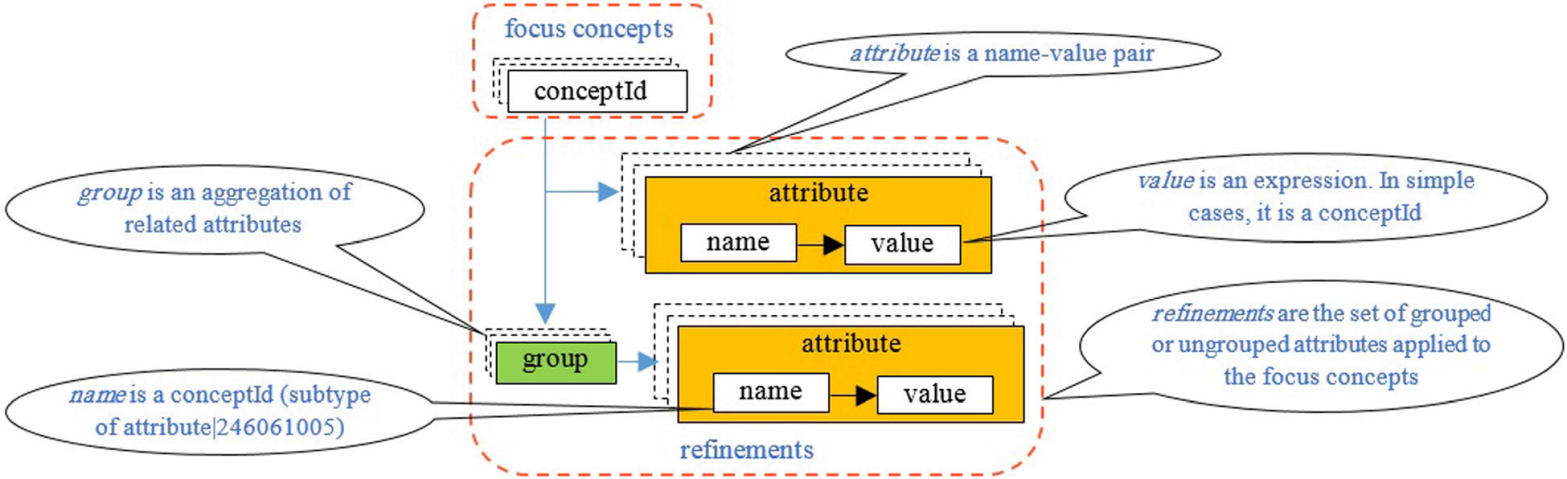
Simple post-coordinated concept (expression) structure

In addition, the example in Fig. 1 describes the idea for *removal of an ovarian structure using a laser device*:

Example 3

===*71388002* |*procedure*|*: 405815000*|*procedure device*| *= 122456005* |*laser device*|*,*

*260686004* |*method*| *= 129304002* |*excision - action*|*,*

*405813007* |*procedure site - direct*| *= 15497006* |*ovarian structure*|

The word *and* is represented by the comma, meaning intersection or conjunction.

(3) A more complicated form of refinement is achieved by *attribute groups* (see Fig. 1). Grouping related attributes avoids ambiguities in complex expressions. There is no limit on the number of groups and the number of attributes in each group. The following example describes a post-coordinated concept: *salpingo-oophorectomy, with laser excision of the right ovary and diathermy excision of the left fallopian tube*.

Example 4

**Fig. 2:**
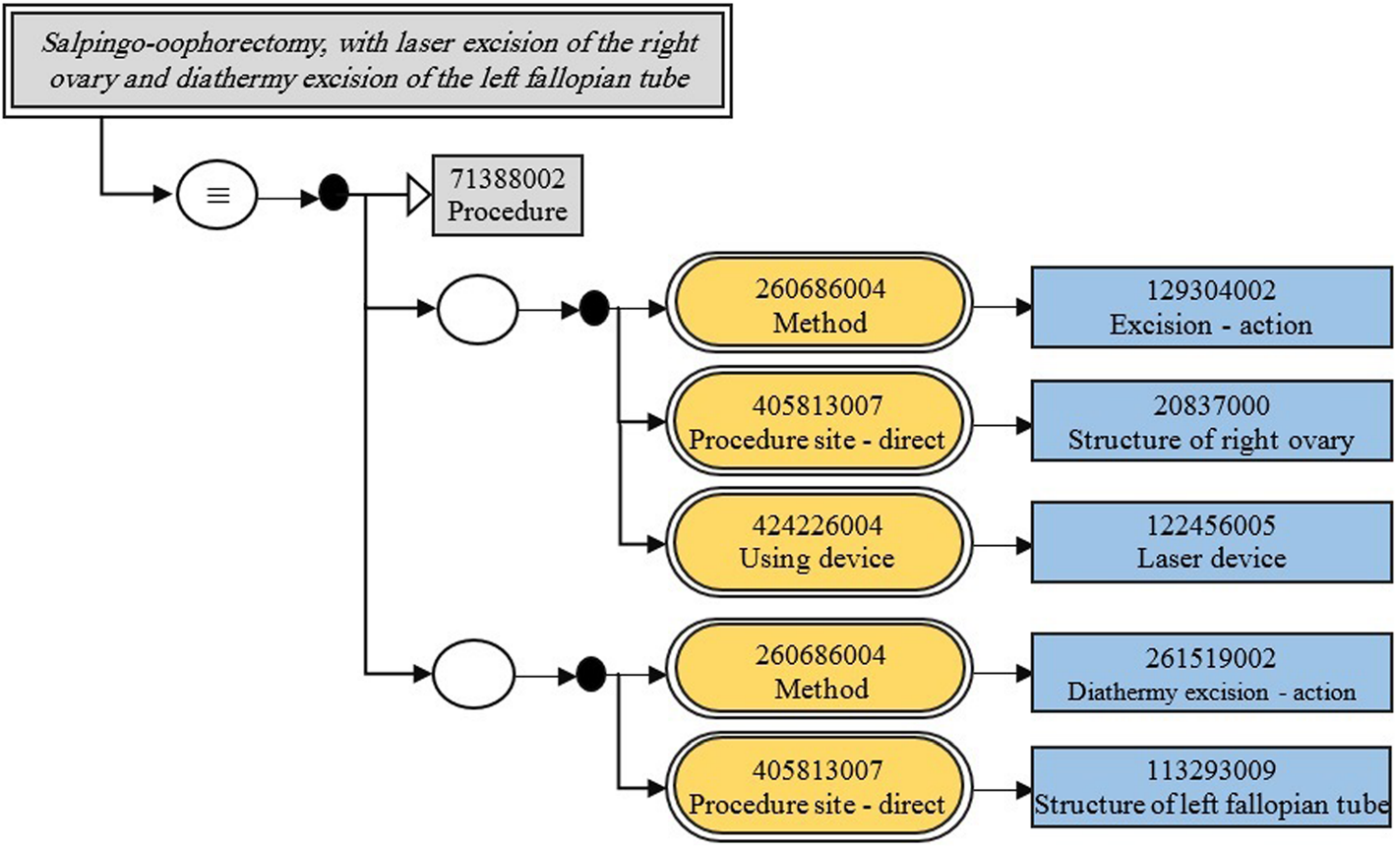
Example 4 using SCT diagramming guidelines

As this example represents complex semantics, it will be illustrated using SCT diagramming guidelines [52], as seen in Fig. 2.

**Fig. 3:**
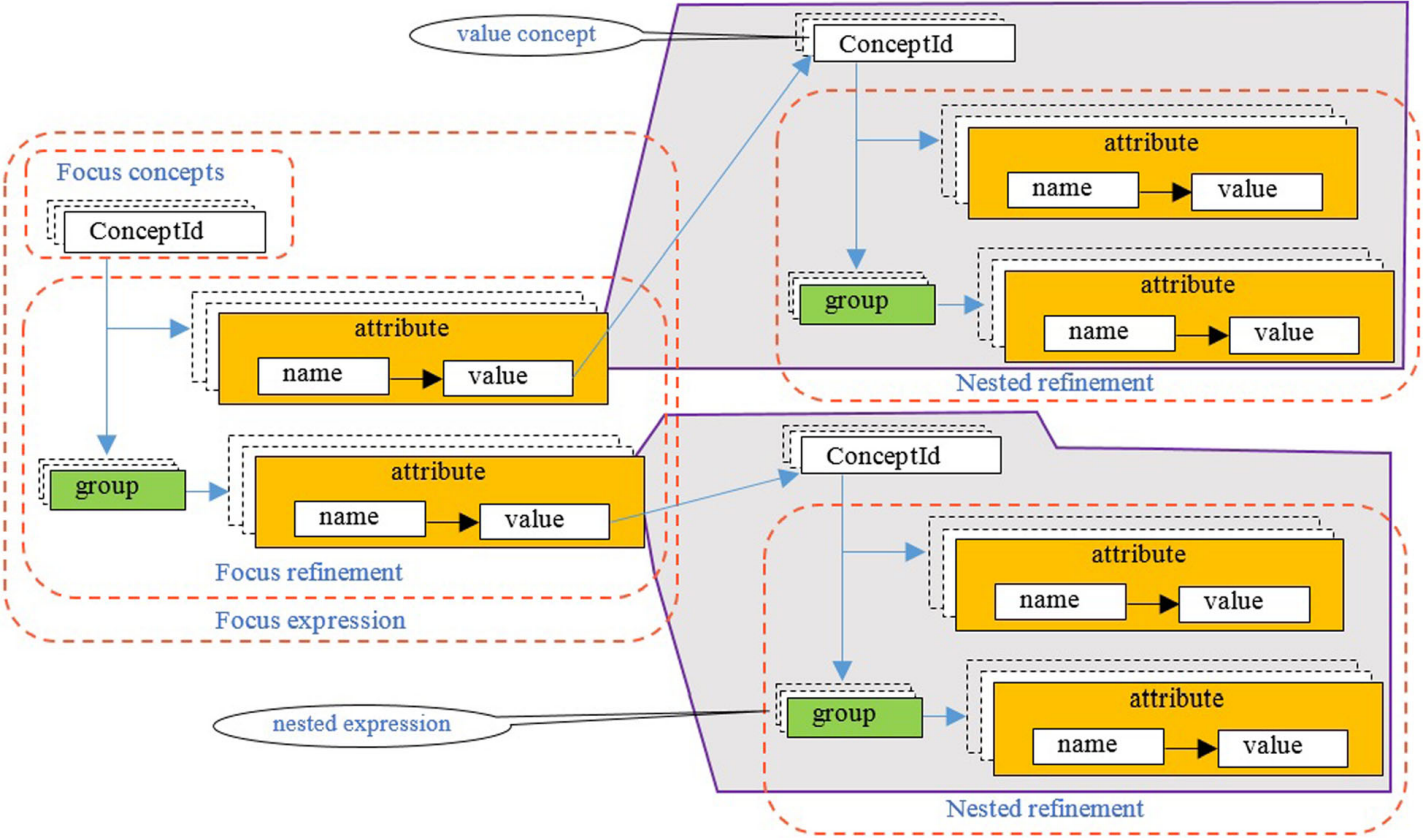
Nested expressions in a complex post-coordinated concept structure

(4) Another more complicated form of refinement is modeled by *nested refinements* (see Fig. 3). Here, a complete expression can be enclosed in double parentheses and used to refine the attribute value of a refining attribute in another expression. This refinement is done by relationship group (RG). For example, the following expression describes a medication product that has a single-dose form, which is both a spray and a suspension:

Example 5

*111613008*|*closed skull fracture with intracranial injury*|*===*

*451000119106*|*closed injury of head*|*, 371162008*|*closed fracture of skull,*

*(116676008*|*associated morphology*|*= 450695007*|*closed traumatic abnormality*|*, 363698007*|*finding site*|*= 128319008*|*intracranial structure*|*),*

*(116676008*|*associated morphology*|*= 20946005*|*fracture, closed*|*, 363698007*|*finding site*|*= 89546000*|*bone structure of cranium*|*)*

Example 5 can be represented in a simplified way using DL syntax without identifiers, as follows:

closed skull fracture with intracranial injury ≡

closed injury of head ⊓ closed fracture of skull ⊓

∃RG (∃associated morphology.closed traumatic abnormality ⊓

∃finding site.intracranial structure) ⊓

∃RG (∃associated morphology.fracture, closed ⊓

∃finding site.bone structure of cranium)

(5) Expressions with *concrete values* have another form. In the previous expressions, attribute values are of concept types, but in current expressions, the attribute value is of primitive types, such as strings, floats, and integers. The expression shown below uses both concept values and concrete values to represent a capsule containing 500 mg of amoxicillin, where 111,115 is the identifier of an attribute.

Example 6

*27658006* |*amoxicillin*|*:*

*411116001* |*has dose form*| *= 385049006* |*capsule*|*,*

*{127489000* |*has active ingredient*| *= 372687004* |*amoxicillin*|*,*

*111115*|*has basis of strength*| *= (111115* |*amoxicillin only*|*:*

*111115*|*strength magnitude*| *= #500, 111115*|*strength unit*| *= 258684004* |*mg*|*)}*

As with Example 5, the following is a simplified representation of Example 6 using DL syntax:

capsule containing 500 mg of amoxicillin ≡

amoxicillin ⊓

∃has dose form.capsule ⊓

∃RG (∃has active ingredient.amoxicillin ⊓

∃has basis of strength.(amoxicillin only ⊓

∃ strength magnitude.#500 ⊓

∃ strength unit.mg))

(6) The final type is the *qualification*, in which a concept is made more specific by applying to the permitted attributes some permitted values or qualifiers like episodicity, severity, and course [3]. The value of the qualifier is mainly a subconcept of *362,981,000*|*qualifier value*|. For example, the concept *periodic fever accompanied by chills* can be modeled as follows:

Example 7

*periodic fever accompanied by chills === 274640006*|*fever with chills*|*: 246456000*|*episodicity*| *= 81591007*|*periodic*|

Depending on the qualifiers used, the resulting concept can be a subtype of the focus concept. For example, *periodic fever accompanied by chills* is subsumed by *fever with chills*, but *known absent Asthma* is not an *Asthma*.

A post-coordinated expression can be a simple expression or a complex expression. As shown in Fig. 1, a simple expression is an expression consisting of one or more conceptIds plus optional refinements. The refinements include any number of attributes, which are expressed as *name–value* pairs and may be applied either independently and/or as parts of groups [4]. The name part is a subclass of concept *246,061,005*|*attribute*|. It is the characteristic, which will be refined. The name part is defined based on SCM [4]. The value is a pre-coordinated conceptId.

Complex expressions are shown in Fig. 3. The main difference between complex and simple expressions is the value of the name–value pair. This value can again be a nested expression, for example, *bacterial infectious disease* affecting the *left upper lobe of the lung* and caused by *Streptococcus pneumonia* is expressed as follows:

Example 8

*87628006*|*bacterial infectious disease*|*:*

*246075003*|*causative agent*|*= 9861002*|*streptococcus pneumonia*|*,*

*363698007*|*finding site*|*= (45653009*|*structure of upper lobe of lung*|*,272741003*|*laterality*|*= 7771000*|*left*|*)*

The resulting expressions can be stored for subsequent use in text files, extensible markup language documents, or relational databases [4]. However, an ontology has the features necessary to manage the consistency and flexibility of these expressions because it is based on formal description logic. This logic enables automatic reasoning and powerful analytic capabilities [26]. Expressions may be nested recursively, so there may be further levels of nested expressions with nested refinements.

## Results

### SCT description logic

In this section, we discuss the capabilities of SCT-based DL. SCT is based on a subset of EL++ formalism. EL++ is a restriction of ALC DL. The key axioms supported by EL++ DL [29] and implemented in SCT are represented in Table 1. The semantics of EL++ is defined in terms of interpretations I = (*∆*^*I*^, .^*I*^), where the domain *∆*^*I*^ is a non-empty set of individuals, and the interpretation function, .^*I*^, maps each class name *C* to a subset *C*^*I*^ of *∆*^*I*^ and each role name *R* to a binary relation *R*^*I*^ in *∆*^*I*^. Interpretation *I* is a model of an ontology, *O*, if and only if for each inclusion axiom in *O* the conditions given in the semantics column of Table 1 are satisfied.

**Table 1:** Syntax and semantics of EL++ DL

Name	OWL syntax	Syntax	Semantics
Top	Thing	⊤	*∆* ^I^
Bottom	Nothing	⊥	∅
Atomic class	Class	C	C^I^
Primitive role	Object and data property	R	R^I^
Existential quantification	ObjectSomeValuesFrom	∃R. C	{x ∈ *∆*^I^| ∃y ∈ *∆*^I^ : (x, y) ∈ R ⋀ y ∈ C^I^}
General class inclusion	SubClassOf	C ⊑ D	C^I^ ⊆ D^I^
Role inclusion	SubObjectPropertyOf	R ⊑ S	{x, y ∈ *∆*^I^| (x, y) ∈ R^I^ → (x, y) ∈ S^I^}
Class equivalence	EquivalentTo	C ≡ D (C ⊑ D, D ⊑ C )	C^I^ = D^I^
Conjunction	ObjectIntersectionOf	C ⊓ D	C^I^ ∩ D^I^
Domain restriction	ObjectPropertyDomain	∃R. ⊤ ⊑ C	{x ∈ *∆*^I^| (x, y) ∈ R^I^} ⊆ C^I^}
Range restriction	ObjectPropertyRange	⊤ ⊑ ∀ R. C	{y ∈ *∆*^I^| (x, y) ∈ R^I^} ⊆ C^I^}
Disjointedness	DisjointWith	C ⊓ D ⊑ ⊥	C^I^ ∩ D^I^ = ∅

This set of class constructors is a small subset of DL features, compared to ALC, SHOIN, SROIQ, etc. Some EL++ constructs are not implemented in SCT, including class disjointedness, property equivalence, transitive object properties, universal quantification (ONLY), disjunction (OR), class negation (NOT), and inverse object properties. These constructors are not supported owing to their complexity and high prerequisites in computation power and time.

SCT semantics and expressions are distributed in the form of compositional grammar [9]. To simplify the modeling of SCT expressions in DL and ontology terms, we suggest some mappings between the terminology of compositional grammar and constructs of OWL and DL (see Table 2). These mappings simplify the conversion of SCT expressions into OWL axioms.

**Table 2:** Mapping between the SCT compositional grammar, ontology, and DL operators

SCT compositional grammar	OWL construct	DL construct
expression, subExpression	Axiom	Axiom
sctid = conceptReference = conceptid = term	String	String
numericalValue	Float/integer	Float/integer
Plus	objectIntersectionOf	⊓
definitionStatus (<<<, ===)	subClassOf, equivalentClasses	⊑, ≡
focusConcept	Class	*C*
refinement	some, objectIntersectionOf	∃, ⊓
attributeGroup	objectIntersectionOf	⊓
attributeSet	objectIntersectionOf	⊓
attributeValue	Data property, object property	Roles
expressionValue	objectIntersectionOf	⊓

### Steps for building an SCT OWL 2 upper-level ontology

**Fig. 4:**
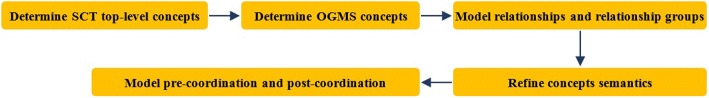
SCTO development methodology

Medical terminologies have so many explicitly defined relationships between each other. In addition, there are many implicit pieces of information that can be inferred from these relationships. Modeling SCT semantics in the form of an ontology is better than using a relational data model because ontologies support consistency checking, and ontology reasoners can discover hidden knowledge [53]. An ontology has a dynamic nature, allowing new information to be added and existing information to be updated in a consistent way. The open world assumption facilitates the flexibility of SCT maintenance. The 2015 SCT version has 19 top-level concepts. To understand what each top-level concept means, we studied their children and read the SCT documentation concentrating on SCM and component structures [3, 30]. Moreover, universals or classes of BFO 2.0 and OGMS were studied to define the equivalences and subsumptions between SCT and OGMS [42]. As shown in Fig. 4, this study builds the SCTO using the following steps:

1. determine the SCT concepts that will be modeled in the ontology;
2. determine the locations (universals) in OGMS where the SCT concepts will be modeled, either as equivalences or subsumptions;
3. solve the problem of relationship and relationship group modeling;
4. use the modeled relationship to add a set of axioms to refine the defined concepts in step 1; and
5. specify how the ontology can model pre-coordination, post-coordination, and SCT constraints.

These steps are discussed in the following subsections.

#### Determining SCT top-level concepts

The 19 SCT concept hierarchies are organized into three main types: *object*, *value*, and *miscellaneous*. The main relationship in SCT is *116,680,003|is a|*, which organizes all concepts in a tree with one parent named *138,875,005|SNOMED CT Concept|*. Other relationships between concepts are formally managed by SCM, and they support the creation of the SCTO axioms. These relationships are represented as *object*-*attribute*-*value*. The *object* is the domain of the relationship and takes values from the hierarchies: *|clinical finding|*, *|procedure|*, *|observable entity|*, *|event|*, *|staging and scales|*, and *|specimen|*. The *value* is the range of the relationship and takes values from the hierarchies: *|body structure|*, *|organism|*, *|substance|*, *|pharmaceutical product|*, *|physical object|*, *|physical force|*, and *|environment or geographical location|*. The *attribute* is the name of the relationship and takes names from the hierarchies: *|qualifier value|*, *|record artifact|*, and *|linkage concept|*.

Organization of attributes in hierarchies enhances the semantics of the resulting ontology. For example, the attribute (property or relationship) |*associated with*| has three subtypes: |*after*|, |*due to*|, and |*causative agent*|. In addition, for each of the 19 hierarchies, SCM identifies its applicable attributes and values. For example, *|clinical finding|* is modeled with 16 attributes and 35 values [3], and any concept subsumed by *|clinical finding|* behaves in the same way. Our priority is to maintain all SCT content in the resulting ontology. However, some of the SCT top-level concepts are ambiguous, such as *Social Context, Situation with Explicit Context*, and *Special Concept*. These three concepts cannot be subsumed to BFO universals; therefore, they have no ontological relevance, and these concepts are not added in the SCTO. The other 16 concepts *are* modeled in the SCTO.

#### Determining the OGMS concepts

**Fig. 5:**
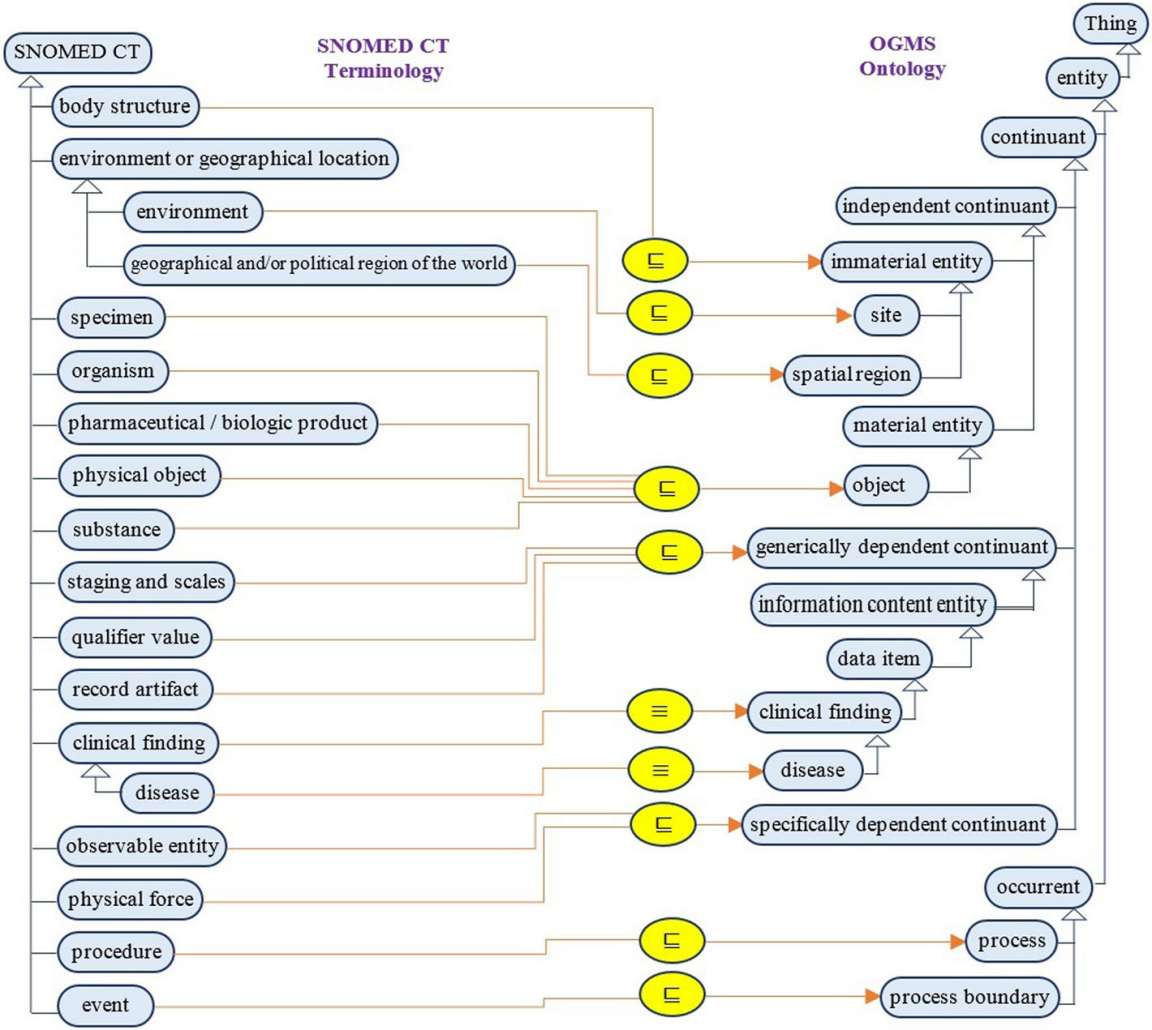
Mapping of OGMS concepts to SCT concepts

This step determines the mapping between SCT top-level concepts and the OGMS ontology universals [44]. Fig. 5 shows how the equivalences (≡) and subsumptions (⊑) are achieved. These concepts are represented in another way in Fig. 5. The first column is the SCT concept; the second column is the OGMS classes; and the last column is the type of mapping. To preserve the logical appearance of SCT hierarchies, we prefer to add one class called *SNOMED CT Concept* ⊑ *entity*. All of the mapped concepts in Fig. 5 are sub-classes of *SNOMED CT Concept* as well.

The *Linkage Concept* has no medical meaning, but it is critical for modeling SCT relationships. As we will see in the next section, SCT relationships cannot be directly modeled as ontology object properties. As a result, this concept is added as a subclass of *SNOMED CT Concept* only. All concepts have a textual definition collected from SCT documentation [3]. All ontological SCT classes have identifiers with the format *SCTO_conceptId,* where *conceptId* is the SCT assigned conceptId for that concept. For example, *SCTO_123037004* is the identifier of the *body structure* concept.

#### Modeling SCT relationships and relationship groups

We classify SCT relationships into two main types: (1) classification relationships implemented by the *116,680,003|is a|* relationship, and (2) other attributes defined as sub-concepts of *410,662,002|concept model attribute|.* The IS_A relationship must not be implemented as an explicit IS_A object property. If source concept C1 is connected with destination concept C2 by a relationship with *typeId = 116,680,003*, then this relationship is mapped as C1⊑ C2 in the ontology. The Concept *definitionStatusId* from the concept table determines if the concept is defined by ⊑ (i.e. primitive) or by ≡ (fully defined). The IS_A relationship has a special nature: it is always ungrouped, so *relationshipGroup = 0*. Moreover, the other attributes have only one value, such as *characteristicTypeId = 900,000,000,000,011,006* and *modifierId = 900,000,000,000,451,002*. As a result, there is no need to repeat these data for all concepts. In order to implement other properties, we have two options. The first is to model SCT attributes in the *410,662,002|concept model attribute|* hierarchy directly as object properties. This option is straightforward, and facilitates the subsequent post-expression definition. However, much of the information about the relationships listed in the relationships file will be lost.

Concerning the second option, in order to preserve this information, we have two ways to put it into practise. Let us examine the first. In OWL 2,^6^ properties (object or data) are binary relations, and OWL 2 and DL do not support the modeling of properties of properties (i.e. N-ary properties) [3]. There is a critical problem in modeling SCT relationships concerning how to describe the instances of relationships. Using an N-ary relationship–modeling process can solve this. For example, the relationship *56,265,001*|*heart disease*| *363,698,007*|*finding site = 80,891,009*|*heart structure*| also has *relationshipId = 2,034,997,023*, *moduleId = 900,000,000,000,207,008*, *typeId = 363,698,007*, *characteristicTypeId = 900,000,000,000,011,006*, *modifierId = 900,000,000,000,451,002, relationshipGroup = 0,* and *active = 1*.

**Fig. 6:**
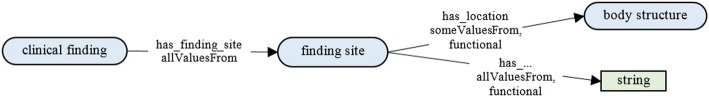
Modeling of N-ary properties

This information must be preserved, because these attributes are important. For example, the active property helps with backward compatibility between SCT versions. We propose two ways to handle this modeling problem. One solution is to represent the relationship as a class (i.e. reification of the property) rather than a property, and create *n* new properties to represent an N-ary relation. For instance, the following example is modeled in Manchester syntax (*http://**www.w3.org/TR/owl2-manchester-syntax/*) where we use the concept terms and not the conceptIds to facilitate readability (Fig. 6).

Class: *‘heart disease’*

SubClassOf:

*‘cardiac finding* *AND* *disorder of mediastinum* *AND* *disorder of cardiovascular system’* AND

( *has_finding_site* ONLY *‘finding site’* )

ObjectProperty: has_finding_site

Domain: *‘clinical finding’*

Range: *‘finding site’*

ObjectProperty: has_location

Domain: *‘finding site’*

Range: *‘body structure’*

Characteristics: Functional

class: *‘finding site’*

SubClassOf:

*‘concept model attribute’* AND (has_location SOME *‘heart structure’* ) AND (has_relationshipId ONLY *“2034997023”* ) AND (has_moduleId ONLY *‘900000000000207008’* ) AND (has_ typeId ONLY *“363698007”* ) AND (has_ characteristicTypeId ONLY *‘900000000000011006’* ) AND (has_ modifierId ONLY *‘900000000000451002’* )

DataProperty: has_...

Domain: *‘finding site’*

Range: xsl:string

Characteristics: Functional

This way of modeling has important advantages. The relationship hierarchies are modeled in the same way as in SCT terminology, and all information distributed in SCT is preserved. However, the modeling of N-ary relationships has many limitations; see the SNOMED CT Technical Implementation Guide [3] for details.

**Fig. 7:**
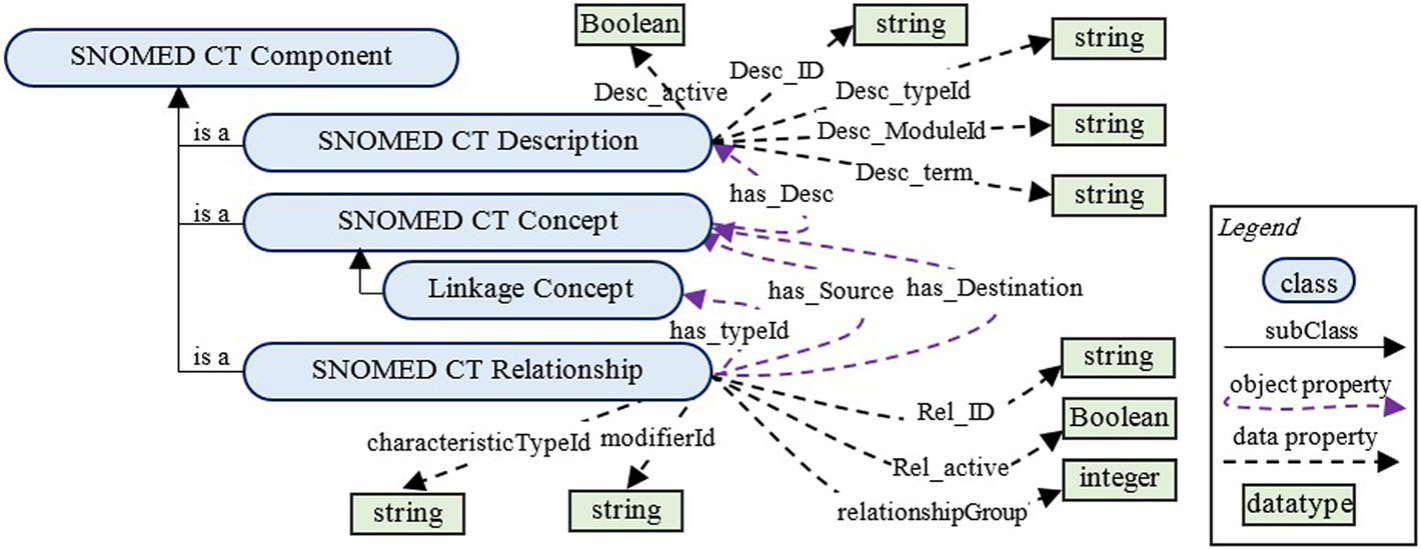
Relationships and descriptions modeling

The second way is to add a top-level concept named “SNOMED CT component” with three subclasses: *SNOMED CT Concept*, *SNOMED CT Description*, and *SNOMED CT Relationship* (see Fig. 7). The instances of *SNOMED CT Description* store the data in a description table for each concept using data properties. The instances of *SNOMED CT Relationship* will store relationship fields and connect SCT concepts. Many properties and inverse properties are not presented in Fig. 7 to simplify readability. Moreover, restrictions such as allValuesFrom, someValuesFrom, and functional are not represented. The modeling problem represented in Fig. 6 can be represented in Fig. 7 in a more formal and straightforward way.

##### Modeling of relationship groups

**Fig. 8:**
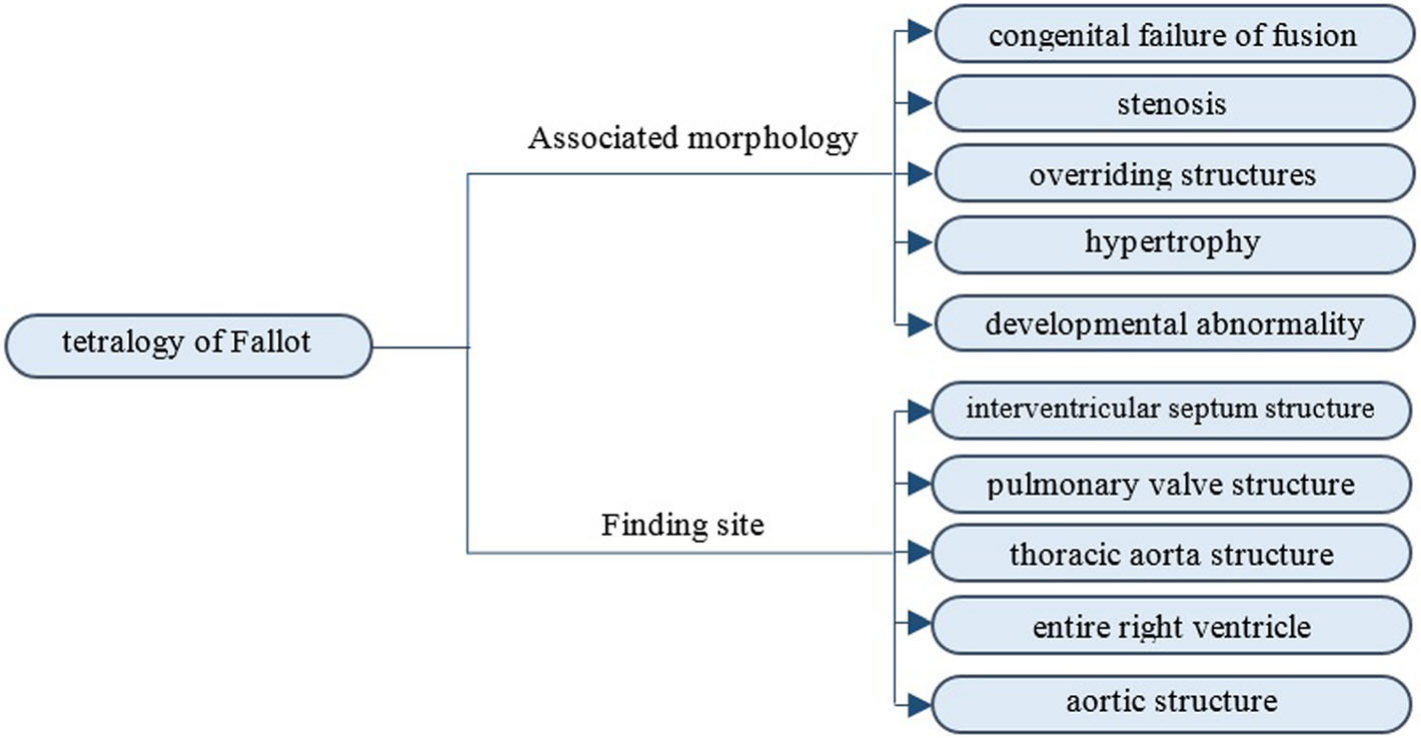
Modeling tetralogy of Fallot without relationship groups

A relationship group combines an *attribute–value pair* with one or more other *attribute–value pairs* (i.e. roles) to add clarity to concept definitions [3]. The purpose of relationship groups is to indicate that certain roles must go together, but their ordering is not required. The RGs add clarity to |*Clinical finding*| concepts by multiple |*Associated morphology*| and |*Finding site*| attributes, and add clarity to |*Procedure|*, which requires multiple |*Method*| and |*Procedure site*| attributes. Without relationship groups, class semantics is not correct. For example, the concept *86,299,006|tetralogy of Fallot|* is modeled without RGs, as shown in Fig. 8, where the five IS_A relationships are not modeled for simplification.

**Fig. 9:**
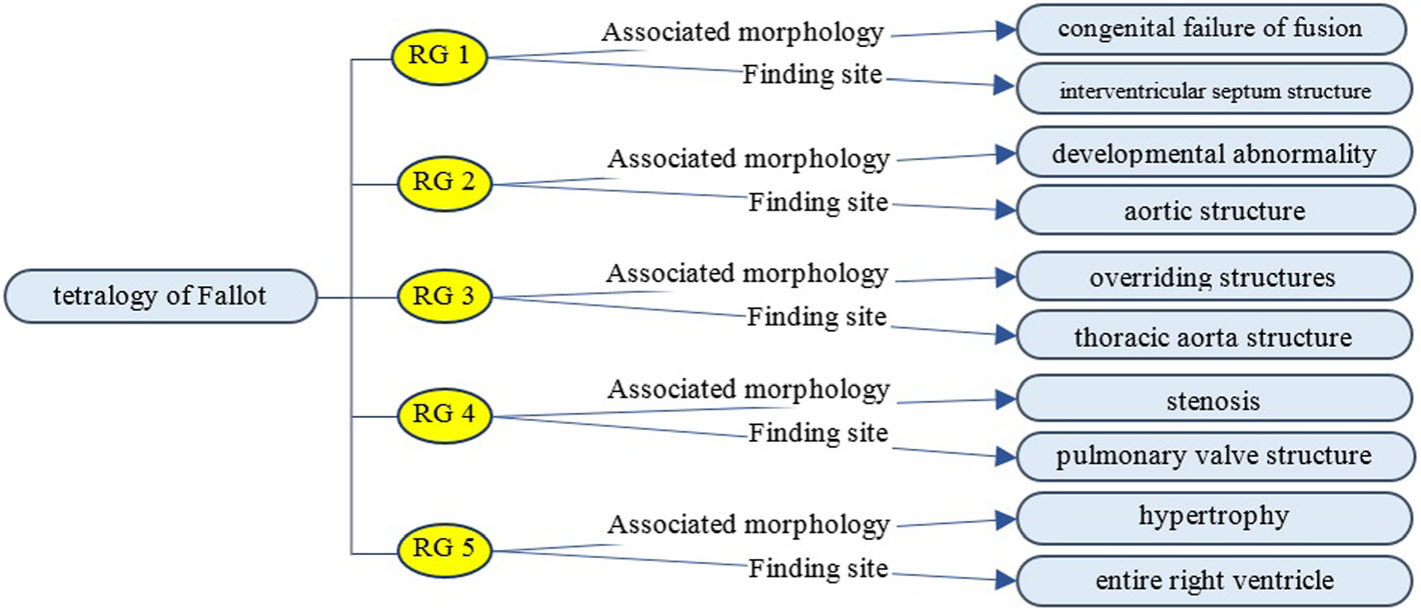
Modeling tetralogy of Fallot with relationship groups

*Tetralogy of Fallot* is a disorder of the heart, which is characterized by five anatomic abnormalities, including a defect at the ventricular septum, stenosis at the pulmonary valve structure, overriding thoracic aorta, and hypertrophy at the right ventricular structure. Relationship definitions in SCT are able to relate tetralogy of Fallot to where these abnormalities are found using the “finding site” relations, and indicate what abnormality it is by using the “associated morphology.” However, there is some confusion because of the many ways one can order the relationships together (see Fig. 8). RGs can solve this problem by grouping an associated morphology with its specific finding site. This process is shown in Fig. 9.

In the 2015 SCT version, there are 1,480,359 relationships defined with relationship groups (47.85%), i.e. with no *quantifiers* and IS_A relationships, and *RelationshipGroup* ≠ 0. *Finding Site* (20.97%), *Associated Morphology* (20.73%), and *Method* (20.82%) are the ones that use RGs the most.

Attribute–value pairs are represented in DL using “exists-restrictions,” denoted (∃*R*. *C*), where *R* is the relationship’s or attribute’s name, and *C* is the expression representing the value concept. In DL, we can use braces, {}, as a symbol for role groups in order to represent the fact that two “exists-restrictions” should occur together. For example, the previously modeled concept *closed skull fracture with intracranial injury* can be normalized in DL notation as follows:

Example 9

≡451000119106 ⊓ 371162008 ⊓

{∃116676008. 450695007 ⊓ ∃363698007. 128319008} ⊓

{∃116676008. 20946005 ⊓ ∃363698007. 89546000}

where the braces are used to model relationship groups.

Many reasoners support inference rules: for instance, Pellet supports OWL 2 profiles, including OWL EL [26]. In the SCTO, the authors added an SWRL rule to bind relationships that have the same relationshipGroup number, as follows:

*Relationship_relationshipGroup(?r1, ?n1), Relationship_relationshipGroup(?r2, ?n2), equal(?n1, ?n2) -> Grouped_Relations(?r1, ?r2)*

Many reasoners like Hermit and Pellet support rule reasoning [26]. The SCTO facilitates the creation of ontology groups. For example, the previous concept in Example 9 can be modeled in the SCTO as follows (see Fig. 7):

Class: *‘closed skull fracture with intracranial injury’*

EquivalentTo: *‘closed injury of head’* **and** *‘closed fracture of skull’* **and**

{(IsSourceOf some (((Relationship_destinationId some *‘intracranial structure’*) and (Relationship_typeId some *‘finding site’*)) and Grouped_Relations exactly 1 ((Relationship_destinationId some *‘closed traumatic abnormality’*) and (Relationship_typeId some *‘associated morphology’*))))

} **and**

{(IsSourceOf some (((Relationship_destinationId some *‘bone structure of cranium’*) and (Relationship_typeId some *‘finding site’*)) and Grouped_Relations exactly 1 ((Relationship_destinationId some *‘fracture, closed’*) and (Relationship_typeId some *‘associated morphology’*))))

One problem to solve with role grouping is the redundancy of expressions. Many authors proposed rules to eliminate redundancy [54]. The redundancy prevention axioms include (1) repeating ungrouped exists-restrictions (**∃R.C** ⊓ {**∃R.C** ⊓  ∃ S. D} = {**∃R.C** ⊓  ∃ S. D}), (2) repeating a group with more general classes ({**∃R.C**_**1**_ ⊓  ∃ S. D} ⊓ {**∃R.C**_**2**_ ⊓  ∃ S. D} = {**∃R.C**_**2**_ ⊓  ∃ S. D} when **C**_**1**_ ***⊒*** **C**_**2**_), and (3) repeating an exists-restriction with more general classes in the same group ({**∃R.C**_**1**_ ⊓  **∃ R.C**_**2**_ ⊓  ∃ S. D} = {**∃R.C**_**2**_ ⊓  ∃ S. D} when **C**_**1**_ ***⊒*** **C**_**2**_), where *R, S* are relationships (i.e. object properties) and **C**_***i***_ represents a concept expression. All these axioms can be modeled in the SCTO in a straightforward way.

#### Refinement of concept semantics

One of the most important reasons for implementing SCT as an ontology is the ability to restrict semantics of concepts and relationships. In this section, we provide some examples from the added semantics for the SCTO. First, we assert that a class cannot be a subclass of more than one top-level hierarchy by the following axiom:

DisjointClasses : *'pharmaceutical / biologic product' 'physical force' 'linkage concept' 'environment or geographical location' 'qualifier value' 'observable entity' 'record artifact' procedure, event specimen substance 'staging and scales' 'physical object' 'clinical finding' organism*

**Fig. 10:**
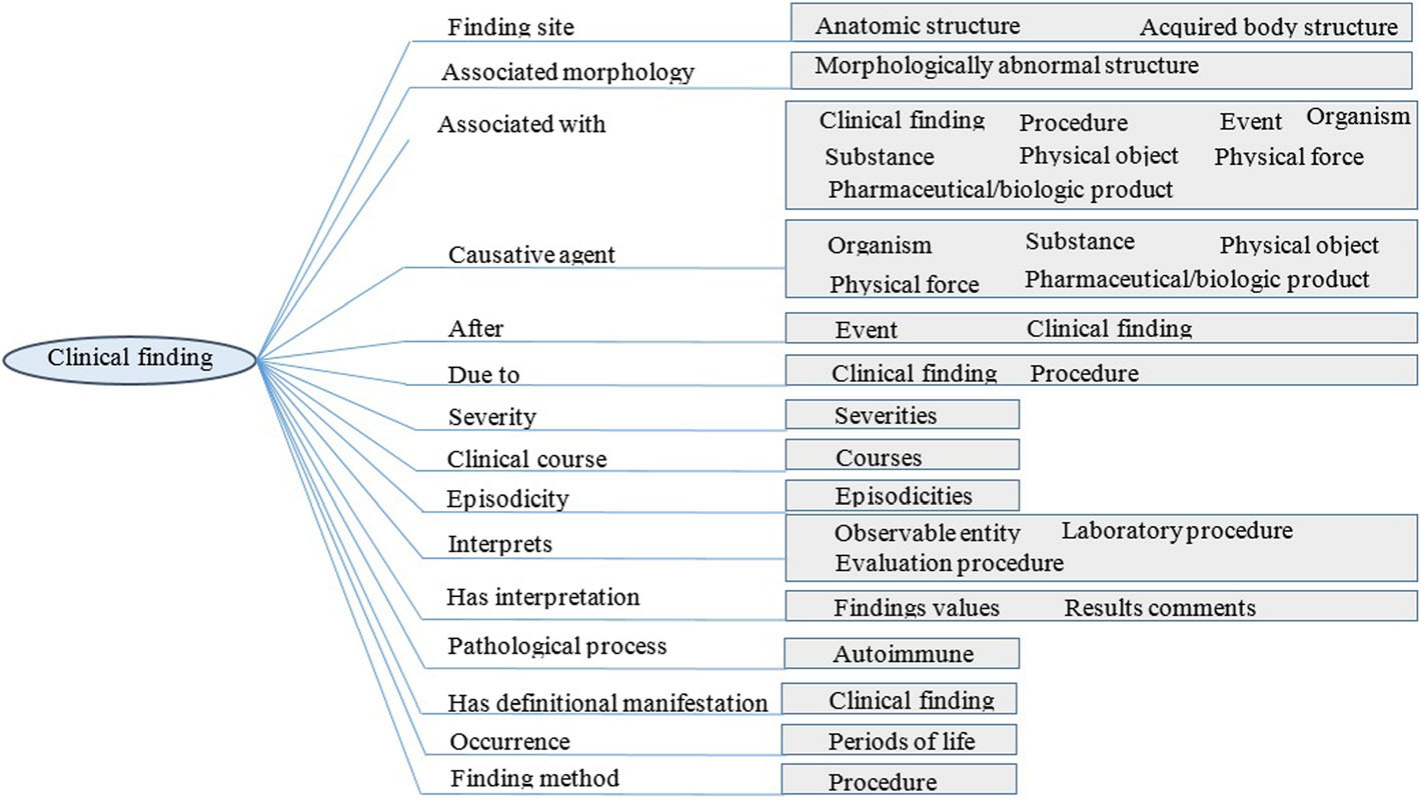
SCT concept model for the clinical finding concept. Adapted from the SCT concept model [3]

Secondly, we try to convert the SCM semantics into axioms. The SCT documentation lists rules for permissible defining attributes for individual hierarchies and the permissible domains for each attribute, but these rules are not computable. Using the SCTO, these rules can be mapped to active axioms and SWRL rules. As a result, the ontology can preserve the consistency of SCT and facilitate its maintenance process. These axioms also support the checking of attribute ranges. Due to space restrictions, we give one example here, and the SCTO on BioPortal at *https://bioportal.bioontology.org/ontologies/SCTO* implements all of these axioms. The clinical finding class is defined in the SCM shown in Fig. 10. The semantics required by the model in Fig. 9 to implement the clinical finding term in Fig. 10 can be implemented as 15 disjunction (*or*) connected OWL axioms (axiom_1_ or … or axiom_15_), one for each branch of Fig. 10. The axiom for the *finding site* of Fig. 10 can be represented as.

axiom _1_ ≡ (IsSourceOf some ((Relationship_destinationId some ( *'acquired body structure'* or *'anatomical structure'* )) and (Relationship_typeId some *'finding site'* )))

All of the other classes are handled in similar ways in the OWL ontology of the SCTO.

#### Modeling of pre-coordination and post-coordination

As discussed before, concept expressions in SCT can be:

- concept name ***C*** as primitive concept (*C* ⊑ *C*_*i*_) or defined concept (*C* ≡ *C*_*i*_);
- conjunctions of concept names: *C*_1_ ⊓ *C*_2_… ⊓ *C*_*n*_;
- conjunction of concept names with exists-restrictions and grouped exists-restrictions: *C*_1_ ⊓ *C*_2_… ⊓ *C*_*n*_ ⊓  ∃ *R*. *C*… ⊓ {∃*R*. *C* ⊓ …} ⊓ …

These types can easily be built by using SCTO classes, object properties, and data properties. The first two types are direct subsumptions, equivalences, and conjunctions of already existing classes, as shown before in Example 2. The third type is the most complex, so we are going to give an example.

Example 5 is similar to the previously modeled Example 9. Example 1 can be modeled as follows:

Class: *‘Diabetes mellitus’*

SubClassOf: *‘disorder of glucose metabolism’* **and** *‘disorder of endocrine system’* **and**

(IsSourceOf some ((Relationship_destinationId some *‘structure of endocrine system’*) and (Relationship_typeId some *‘finding site’*)).

Examples 3, 4, and 7 are modeled in a similar way. Let us model the most complex one (Example 4) as follows:

Class: *‘salpingo-oophorectomy, with laser excision of the right ovary and diathermy excision of the left fallopian tube’*

SubClassOf:

*{*

*‘procedure’ and* (IsSourceOf some ((((Relationship_destinationId some *‘excision - action’*) and (Relationship_typeId some *‘method’*)) and Grouped_Relations exactly 1 ((Relationship_destinationId some *‘structure of right ovary’*) and (Relationship_typeId some *‘procedure site - direct’*))) and Grouped_Relations exactly 1 ((Relationship_destinationId some *‘laser device’*) and (Relationship_typeId some *‘using device’*)))

*}* ***and***

*{*

*‘procedure’ and* (IsSourceOf some (((Relationship_destinationId some *‘diathermy excision - action’*) and (Relationship_typeId some *‘method’*)) and Grouped_Relations exactly 1 ((Relationship_destinationId some *‘structure of left fallopian tube’*) and (Relationship_typeId some *‘procedure site - direct’*))))

*}*

In this example, we repeated the class *Procedure* in both axioms to refine the selected concepts to be of the procedure type. Moreover, in the first group of this post-coordinated expression, we link three relationships with one relationship group. Due to space restrictions, we will not model the other examples, but they can be modeled in the same way.

#### The resulting SCTO

The overall result is a regular, standard, and uniform ontology more consistent than the SCT original ontology. The ontology is created with an OWL 2 format, which makes it easier to use, query, and ensure quality as the basis for the software. We focused on creating the SCTO’s TBOX. Moreover, we designed the top-level classes only based on the BFO and OGMS ontologies. There are 14 subsumption mappings (82.35%), two equivalence mappings (11.77%), and concepts with no equivalence (5.88%). All of the 19 SCT hierarchies have been modeled in the SCTO under suitable BFO and OGMS universals, except three concepts: (1) *243796009|situation with explicit context*, which can be modeled with other existing classes; (2) *48176007|social context*, which has ambiguous semantics and cannot be mapped to any universal; and (3) *370115009|Special Concept*, which had not appeared in the SCM. The resulting ontology has no instances (i.e. ABOX) because the instantiation takes place for each individual’s medical record. Actually, TBOX alone equals an ontology, but TBOX + ABOX is a knowledge base. Knowledge bases are used in specific systems for specific purposes, but they are outside the scope of this paper. The SCTO contains only the SCT top-level concepts, and focuses on modeling the semantics of these concepts in the form of OWL2 axioms. Fig. 5 depicts the upper-level hierarchy of the SCTO based on OGMS at the topmost level.

The creation of all the SCT hierarchies can be automated by using the existing OWL API and reasoners such as Pellet, RacerPro, Hermit, etc. The SCTO implements the SCT formal and standard concept model and solves the modeling problems of pre-coordinated and post-coordinated concepts. OWL ontologies support the creation of more complex expressions built recursively from previously defined classes and properties using constructors provided by the ontology’s language and logic. We used Protégé 5 to implement the SCTO.

Table 3 lists SCTO object properties, and their definitions, domains, and ranges. There are only eight object properties in the SCTO. By using only these properties, we have the ability to model SCT expressions, relationship groups, and constraints, as shown previously. The SCTO contains 20 data properties. Table 4 shows some examples from these properties.

**Table 3:** Object properties of the SCTO

Property	Definition	Domain	Range
Has_description	Determines a description for a class.	SNOMED CT Concept	SNOMED CT Description
IsDescriptionOf	Is the inverse of Has_description.	SNOMED CT Description	SNOMED CT Concept
Relationship_destinationId	Identifies the class that is the destination of the relationship. Set to an identifier of a concept in the Concept file.	SNOMED CT Relationship	SNOMED CT Concept
Relationship_sourceId	Identifies the source concept of the relationship. Set to an identifier of a concept in the Concept file.	SNOMED CT Relationship	SNOMED CT Concept
Relationship_typeId	A concept enumeration value from the metadata hierarchy that identifies the semantic type of the relationship. It is a subtype of 410,662,002 |Concept model attribute|.	SNOMED CT Relationship	Linkage Concept
IsSourceOf	Determines the source of a relationship. It is the inverse of Relationship_sourceId.	SNOMED CT Concept	SNOMED CT Relationship
IsDestinationOf	Determines the destination of a relationship. It is the inverse of Relationship_destinationId.	SNOMED CT Concept	SNOMED CT Relationship
Grouped_Relations	Used to explicitly determine the grouped relationships	SNOMED CT Relationship	SNOMED CT Relationship
Total number of object properties			8

**Table 4:** Data properties of the SCTO

Property	Definition	Domain	Range
Concept_Id	The unique SNOMED CT Identifier for this Concept.	SNOMED CT Concept	String
Description_term	The description’s text value, represented in UTF-8 encoding.	SNOMED CT Description	String
Description_typeId	Identifies whether the description is an FSN, synonym, or other description type.	SNOMED CT Description	String
Relationship_relationshipGroup	Groups together relationship versions that are part of a logically associated relationship group.	SNOMED CT Relationship	Integer
Relationship_active	Specifies whether the relationship’s state is active or inactive.	SNOMED CT Relationship	Boolean
Relationship_characteristicTypeId	A concept enumeration value that identifies the characteristic type of the relationship.	SNOMED CT Relationship	String
**…**			
**Total number of data properties**			**20**

Building a top-ontology–based SCTO has many benefits for new medical informatics. The resulting ontology is based on OGMS, which in turn uses BFO as its top-level ontology, a feature providing the unified and global semantics of SCT concepts. This way, SCT can be integrated with other ontologies, such as Logical Observation Identifiers Names and Codes (LOINC) and GO, etc. [40, 55], in a unique, logically, and medically consistent and applicable way. The SCTO can be integrated into the EHR healthcare environment with the Health Level 7 Reference Information Model to support semantic interoperability. Semantic queries using Simple Protocol and RDF (Resource Description Framework) Query Language (SPARQL) and Semantic Query-Enhanced Web Rule Language (SQWRL) can use reasoner inference capabilities to retrieve hidden information besides explicit information. The ontology can be used in clinical decision support systems as a means to determine levels of similarities and relationships between compared concepts. The SCTO is freely available on BioPortal, a web portal that provides a uniform mechanism to access biomedical ontologies and terminologies provided in different representation formats, including OBO and OWL. The following URL provides direct access to the SCTO on BioPortal: *https://bioportal.bioontology.org/ontologies/SCTO*, where it can be browsed, searched and visualized.

The SCTO is considered a first step towards a complete SCT OWL ontology. Table 5 provides a summary of the SCTO in terms of several statistical and quality-control metrics. The ontology currently incorporates 304 classes, 2400 axioms, 8 object properties, 20 data properties, and 1555 annotations. Each concept has a unique identifier with the format *SCTO_ConceptId*, such as *SCTO_90,708,001* for the *kidney disease* class. Each class and property has a standard definition from SCT documentation.

**Table 5:** SCTO metrics

Metric	Value
Number of classes	304
Axioms	2400
Object properties	8
Data properties	20
Maximum number of parents	3
Average number of siblings	3.62
Maximum number of children	66
Average number of children	3
Classes with more than 25 children	1
Classes with a single child	25
Maximum number of siblings	66
Subclass axioms	330
Annotations	1555
Maximum depth	8

*Consistency checking* is syntactic-level evaluation. In order to confirm that the SCTO is consist and error-free, HermiT (version 1.3.8), Pellet, and FaCT++ reasoners were used with the Protégé 5 editor, and they revealed no discrepancies in the ontology. Moreover, the online tool Ontology Pitfall Scanner! (OOPS!^7^) helped to detect some of the most common pitfalls appearing when developing ontologies. We ran OOPS! on the SCTO to ensure it is free of these pitfalls.

## Discussion

We used SCT terminology to build some medical applications, and we experienced its current limitations discussed previously. We used SCT to build DDO [56], which is a diabetes diagnosis ontology, and DMTO [57], which is a diabetes mellitus treatment ontology. The resulting ontology enhances the semantics of SCT. Generally, there are some studies, which tried to enhance the semantics of SCT [31]. However, very few studies have unified its high-level concepts under suitable universals of top-level ontologies, and no studies have implemented the SCT concept model in OWL axioms using these top-level ontologies. Table 6 provides a comparison between these studies according to a set of metrics. In 2015, Souvignet and Rodrigues [58] followed a similar methodology to create a patient safety ontology by mapping the Patient Safety Categorical Structure data model to BFO 2. Héja et al. [32] mapped SCT to the DOLCE upper-level ontology, and they highlighted some ontological errors in SCT. However, they failed to propose an acceptable SCT ontology. As can be seen in Table 6, the SCTO is the most complete ontology, and combines the advantages of all the other studies.

**Table 6:** A comparison between the SCTO and existing SCT studies

The study	Format	The base	Top-level ontology	Description logic	Modeled hierarchies	Publicly available	Size	Can model pre-coordinated concepts	Can model post-coordinated concepts	Handled semantics
Proposed SCTO	Ontology	SCM	BFO and OGMS	EL++	Whole SCT	OWL format	Top-level concepts	Yes	Yes	Complete OWL axioms for all top-level SCT concepts
SCT Perl script [3]	Ontology	No	No	EL++	Whole SCT	Perl script	Low-level concepts	Yes	No	SCT taxonomy
Schulz & Martínez-Costa [31]	Ontology	No	BioTopLite2	OWL DL	Whole SCT	No	Top-level concepts	Yes	No	SCT taxonomy and basic relations
Campbell et al. [20]	Graph database	No	No	No	Whole SCT	No	Low-level concepts	Yes	No	SCT taxonomy
Martínez-Costa and Schulz [59]	Ontology	SCT context model	BioTopLite	OWLDL	Clinical findings	No	Low-level concepts	Yes	Yes	SCT taxonomy and basic relations
Cheetham et al. [60]	Ontology	No	BioTopLite2	OWLDL	Disorders	No	Low-level concepts	Yes	Yes	SCT taxonomy and basic relations
Bodenreider [61]	Ontology	No	No	OWL EL	Disorders, procedures	No	Low-level concepts	Yes	No	SCT taxonomy
Hogan [62]	Ontology	No	BFO	OWL EL	Whole SCT	No	Top-level concepts	No	No	SCT taxonomy
Ochs et al. [63]	Abstraction network	No	No	No	Observable entity	No	Low-level concepts	Yes	No	SCT taxonomy

The immediate application of the resulting ontology is support for shared and unified understanding of SCT concepts and top-level universals. The defined classes have formal definitions based on DL, and they implement the SCM. SCTO supports the construction of distributed EHR systems and clinical decision support systems. It can be utilized in machine learning and natural language processing studies to understand the semantic meaning of medical concepts. This ontology can support information-retrieval applications by providing a vocabulary and a taxonomy that can be used for query expansion as well as semantic searches. As a result, the ontology supports the creation of semantically intelligent clinical decision–support applications. This version of the SCTO ontology has some limitations. It formally models the top-level classes of SCT hierarchy. After populating SCTO ontology with the other classes, relationships, and descriptions, the resulting ontology is expected to be more accurate, smaller in size, and interoperable with other ontologies. The resulting ontology needs to be tested in some real applications to measure its efficiency in the representation of complex semantics. These limitations will be covered in the future studies.

## Conclusions

In this paper, we introduced the SCTO, which is based on the globally approved OGMS, which is in turn based on the BFO 2.0 top-level ontology. The 2015 SCT version’s SCM was implemented in the form of OWL 2 axioms. The authors resorted to EL++ DL, which is supported by SCT. The paper’s main aim is to preserve all information released in SCT files, including concepts, descriptions, and relationships. All possible SCT expressions, including pre-coordinated and post-coordinated expressions, can be implemented using SCTO terminology (classes, object properties, and data properties). The SCTO contains 304 universals (classes and subclasses), 28 properties, 1555 annotations, and 2400 axioms. It is publicly available through BioPortal at *http://bioportal.bioontology.org/ontologies/SCTO/*. The resulting ontology can be used to integrate SCT with other terminologies, such as GO, LOINC, and RxNorm, because its concepts have unified semantics under OGMS ontology universals [40, 55].

In the future, we will use OWL 2 APIs to populate the SCTO with all of the SCT concepts, descriptions, and relationship instances. The SCT population has some tricky logical issues, such as whether or not the SCT terminology concept will be mapped to SCT ontology classes or individuals, and whether or not all classes should have at least one sibling, etc. Moreover, before population, the confusion between pathological structure, disposition, and process needs to be resolved, especially in the *clinical finding*, *procedure*, *event*, and *body structure* hierarchies: these issues will be identified and modeled. After population, ontology consistency checking will be performed, and the SCTO will be used for semantic queries taken from real healthcare environments. Moreover, we will solve the problem of situations that have an explicit context. The resulting ontology can be merged with other ontologies in specific domains, such as GO and LOINC [55]. As a result, SCT coverage will be enhanced.

## Abbreviations

DL: Description logic
EHR: Electronic health record
GO: Gene ontology
LOINC: Logical observation identifiers names and codes
OBO: Open biomedical ontologies
OGMS: Ontology for general medical science
OWL: Web ontology language
RDF: Resource description framework
SCT: SNOMED CT
SNOMED CT: Systematized nomenclature of medicine—clinical terms

## Footnotes

1
*https://www.w3.org/TR/owl2-primer/*

2
*https://www.w3.org/2007/OWL/wiki/EL*

3
*https://tu-dresden.de/ing/informatik/thi/lat/forschung/software/cel*

4
*http://ifomis.uni-saarland.de/bfo/*

5
*http://protege.stanford.edu* */*

6
*https://www.w3.org/TR/owl2-overview/*

7
*http://oops.linkeddata.es* */*
